# Supplementary material for: Working Memory-Related Neurofunctional Correlates Associated with the Frontal Lobe in Children with Familial vs. Non-Familial Attention Deficit/Hyperactivity Disorder
Source: Brain Sci. 2023 Oct 18;13(10):1469. doi: 10.3390/brainsci13101469 (PMC10605263; doi:10.3390/brainsci13101469)
Supplement: Supplementary file 1 [file brainsci-13-01469-s001.zip › brainsci-2647904-supplementary.pdf]

Working Memory-related Neurofunctional Correlates associated with Frontal Lobe in Children with Familial vs. Non-Familial Attention Deficit/Hyperactivity Disorder

Supplementary Materials

Supplementary Table S1. Group comparisons of the n-back task-performance measures (TDC: Typically developing children; ADHD: Attention deficit/hyperactivity disorder; ADHD-NF: Non-familial ADHD; ADHD-F: Familial ADHD; SD: Standard deviation).

TDC vs ADHD				
	TDC [Mean (SD)]	ADHD [Mean (SD)]	t-statistic	*p*
Percentage accuracy				
0-back	88.67 (8.48)	88.38 (8.89)	t(344.438)=1.880 +	0.061
2-back	79.648 (8.07)	80.43 (8.46)	t(360)=1.704	0.089
Mean reaction time				
0-back	891.83 (118.56)	869.21 (112.95)	t(360)=0.779	0.437
2-back	995.86 (115.68)	971.79 (113.98)	t(360)=1.525	0.128
ADHD-F vs ADHD-NF				
	ADHD-F [Mean (SD)]	ADHD-NF [Mean (SD)]	t-statistic	*p*
Percentage accuracy				
0-back	88.87 (9.028)	87.95 (8.78)	t(284)=0.876	0.382
2-back	81.59 (8.75)	79.39 (8.07)	t(284)=2.216	**0.027**
Mean reaction time				
0-back	875.08 (109.08)	863.95 (116.41)	t(284)=0.832	0.406
2-back	972.63 (110.23)	971.05 (117.59)	t(284)=0.117	0.907

+ Levene's test for homogeneity of variance (centered on mean) showed a significant difference in variance between the two groups. Bold p-values represent statistically significant group differences at $\alpha < 0.05$.

Supplementary Table S2. Working memory-related (2-0 condition) voxel-based brain activation group differences (cluster threshold > 1000 voxels, $\alpha \leq 0.001$; TDC: Typically developing children; ADHD: Attention deficit/hyperactivity disorder; ADHD-NF: non-Familial ADHD; ADHD-F: Familial ADHD; B/L: Bilateral).

Brain regions (within the cluster)	Cluster size (in voxels)	*p*	Contrast
Left inferior frontal gyrus (triangular)<br>Left inferior frontal gyrus (opercular)<br>Left precentral	4979	**1.72E-14**	TDC – ADHD
Right superior frontal<br>Right middle frontal	2808	**1.57E-09**	
B/L precuneus<br>Left cuneus	2157	**5.96E-08**	
Right middle occipital<br>Right superior parietal<br>Right inferior parietal<br>Right angular	1050	**2.11E-04**	
B/L postcentral<br>B/L precentral<br>Left rolandic operculum	11094	**1.60E-25**	ADHD – TDC
Right inferior temporal<br>Right middle temporal<br>Right hippocampus	9784	**1.79E-06**	ADHD-F – ADHD-NF

Left superior frontal gyrus, medial orbital<br>Left anterior cingulate cortex, pregenual<br>B/L superior frontal gyrus, medial	11412	**2.38E-07**	ADHD-NF – ADHD-F

Bold p-values represent statistically significant group differences.

Supplementary Table S3. ROI-based activation and laterality analyses (ROI: Region-of-interests; ANOVA: Analysis of variance; M: Mean; SD: Standard deviation; SFG: Superior frontal gyrus; MFG: Middle frontal gyrus; IFG: Inferior frontal gyrus; TDC: Typically developing children; ADHD: Attention deficit/hyperactivity disorder; ADHD-NF: non-Familial ADHD; ADHD-F: Familial ADHD; LI: Laterality index. Post-hoc t-test with Bonferroni corrections).

Measure	One-way ANOVA						Post-hoc independent samples t-test					
	TDC		ADHD-NF	ADHD-F	F(2)	*p*	TDC – ADHD-NF		TDC – ADHD-F		ADHD-NF – ADHD-F	
	Sample	M (SD)	M (SD)	M (SD)			t-statistic	*p*	t-statistic	*p*	t-statistic	p
Left SFG mean activation	1	1.28 (1.79)	1.08 (1.57)	0.75 (1.51)	3.798	**0.023**	t(454)=1.287	0.199	t(438)=3.147	**0.002**	t(284)=1.771	0.078
	2	1.29 (1.63)			4.309	**0.014**						
Right SFG mean activation	1	1.04 (1.61)	0.67 (1.43)	0.61 (1.45)	3.597	**0.028**	t(454)=2.528	**0.012**	t(438)=2.745	**0.006**	t(284)=0.302	0.763
	2	1.06 (1.55)			4.008	**0.019**						
LI SFG	1	0.03 (0.58)	0.11 (0.59)	0.05 (0.56)	0.804	0.448						
	2	0.09 (0.58)			0.466	0.628						
Left MFG mean activation	1	0.78 (1.79)	0.62 (1.50)	0.54 (1.43)	0.854	0.427						
	2	0.95 (1.67)			2.960	0.053						
Right MFG mean activation	1	1.05 (1.86)	0.62 (1.81)	0.61 (1.52)	2.998	0.051						
	2	1.01 (1.83)			2.602	0.075						
LI MFG	1	-0.09 (0.57)	0.03 (0.63)	-0.05 (0.61)	1.728	0.179						
	2	-0.003 (0.61)			0.707	0.494						
Left IFG mean activation	1	1.19 (1.73)	0.51 (1.59)	0.51 (1.42)	9.174	**<0.001**	t(454)=3.665	**<0.001**	t(299.75)=3.851	**<0.001**+	t(284)=-0.018	0.986
	2	1.02 (1.61)			5.474	**0.004**						
Right IFG mean activation	1	1.04 (1.60)	0.42 (1.67)	0.69 (1.73)	5.243	**0.006**	t(454)=4.011	**<0.001**	t(438)=2.222	0.027	t(284)=-1.344	0.180
	2	1.10 (1.58)			6.331	**0.002**						
LI IFG	1	0.03 (0.60)	0.01 (0.69)	-0.05 (0.64)	0.700	0.497						
	2	-0.03 (0.66)			0.332	0.718						

+ Levene's test for homogeneity of variance (centered on mean) showed a significant difference in variance between the two groups

Bold p-values represent statistically significant group differences.

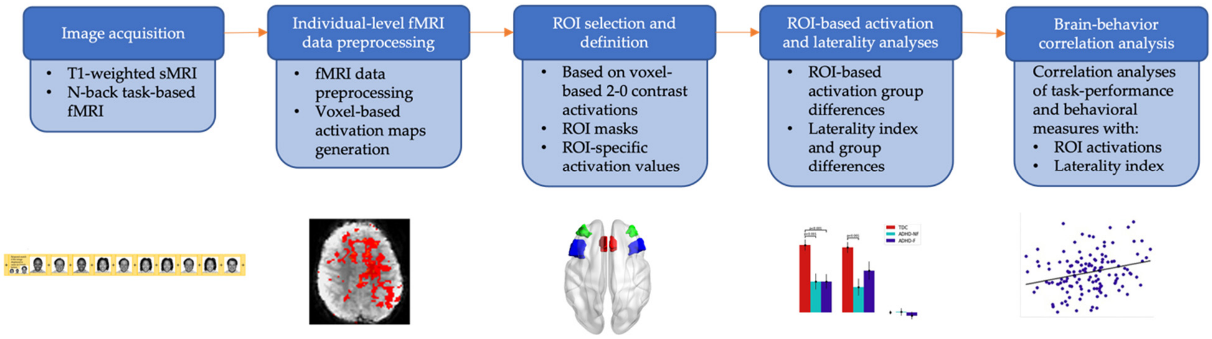

Supplementary Figure S1. Summary of steps in methodology (ADHD: Attention deficit/hyperactivity disorder; ADHD-NF: Non-familial ADHD; ADHD-F: Familial ADHD; ROI: Region-of-interest; fMRI: functional magnetic resonance imaging; sMRI: structural MRI).
